# Echinococcosis: From Parasite–Host Interaction to Rapid Detection

**DOI:** 10.3390/tropicalmed10010001

**Published:** 2024-12-24

**Authors:** Ting Zhang, Zheng Feng

**Affiliations:** 1 National Institute of Parasitic Diseases, Chinese Center for Disease Control and Prevention (Chinese Center for Tropical Diseases Research), National Key Laboratory of Intelligent Tracking and Forecasting for Infectious Diseases, National Health Commission Key Laboratory of Parasite and Vector Biology, WHO Collaborating Center for Tropical Diseases, National Center for International Research on Tropical Diseases, Shanghai 200025, China; zfeng0909@163.com; 2 National Health Commission Key Laboratory of Echinococcosis Prevention and Control, Xizang Center for Disease Control and Prevention, Lhasa 850000, China; 3 The State Key Laboratory of Reproductive Regulation and Breeding of Grassland Livestock, School of Life Sciences, Inner Mongolia University, Hohhot 010010, China

## 1. Introduction

Echinococcosis is one of the most serious and life-threatening parasitic zoonoses worldwide caused by the larvae of the *Echinococcus* genus. The two main types of this disease are cystic echinococcosis (CE) and alveolar echinococcosis (AE), which pose a substantial threat to global public health and graziery [1]. *Echinococcus* completes its life cycle and establishes transmission through two types of hosts. The intermediate hosts are typically mammals like sheep, goats, cattle, and rodents, which become infected by ingesting the eggs. The definitive hosts are carnivorous predators like dogs, wolves, and foxes, which become infected by consuming animal offal containing the larvae. Humans can become accidental intermediate hosts. Upon infection, the parasite larvae reside most commonly in the liver of humans and other mammals [2]. However, the complexity in both *Echinococcus* development and transmission creates great difficulty in prevention and control of the disease. Sensitive and rapid diagnosis is crucial to detect infection at an early stage, assess the prevalence, and take effective prevention and control measures in time. Bolor Bold et al. (contribution 1) evaluated the under-reporting rates in rural Mongolia and found that, due to weakness in diagnosis, treatment, and surveillance, the actual prevalence of CE was eight times higher than that reported. In addition, their results showed the significance and urgent need for early detection, cyst classification, and easily applicable rapid diagnostic tests to face the challenge of echinococcosis management and surveillance in low- and middle-income countries.

So far, the detection of echinococcosis has involved methods such as imaging, serology, autopsy, microscopy, and DNA detection. However, for different animals and humans, the development of diagnostic methods is out of balance and limited, especially for detection in artiodactyls and wild mammals. Nagwa I. Toaleb et al. (contribution 2) reported a novel designed antigen-based sandwich enzyme-linked immunosorbent assay (ELISA) for CE diagnosis in camels with diagnostic efficacy of 96.8%; low cross-reactivity with *Fasciola gigantica* infection and myiasis; and no cross-reactivity with *Eimeria* spp., *Toxoplasma gondii*, *Cryptosporidium* sp., and *Hyalomma dromedarii*. Additionally, a comparable CE positivity rate of 48.7% was found in 587 camels by using the sandwich ELISA, which was comparable to the positivity rate of 46.5% found by postmortem inspection in this study, which showed that the novel serological ELISA test is of medical and veterinary importance in CE detection in camels. Survey reports on echinococcosis in camels are rarely seen. It is worth noting that the novel ELISA found that pulmonary echinococcosis was significantly more prevalent than the hepatic type in the slaughtered camels examined. *Echinococcus* eggs in canine feces are the main indicator and the source of transmission; thus, previous studies in endemic areas demonstrated that deworming dogs with praziquantel is a successful strategy used to control echinococcosis [3]. Several methods exist for the detection of canine echinococcosis, such as the arecoline purgation, copro-ELISA, and copro-PCR approaches, which have greatly promoted epidemiological investigations into canine echinococcosis; however, the use of simple and convenient on-site detection tests in endemic areas is still recommended. Xinyue Lv et al. (contribution 3) developed a new loop-mediated isothermal amplification–lateral flow dipstick (LAMP-LFD) test, providing a straightforward and portable molecular diagnostic tool that can differentiate between *E. granulosus* and *E. multilocularis* using their repetitive genetic sequences with high detection sensitivity (96.05%) and specificity (97.35%). This technique significantly reduces false positives caused by aerosol pollution in the LAMP assay, thus assisting on-site epidemiological investigations into *Echinococcus*.

In some areas like China, CE and AE are highly co-prevalent, and the differentiation between human AE and CE is an important clinical consideration because the two diseases have different pathological outcomes, treatment and management options, and even epidemiological characteristics. Currently, the clinical diagnosis of echinococcosis is mainly based on imaging techniques (ultrasound, computed tomography, and magnetic resonance imaging) which can reveal space-occupying lesions and their location and size in affected organs, but they cannot always identify the species, particularly in early infection with cystic lesions that are small in size. Serology has proved a useful adjunct diagnostic technique. The ELISA technique has been available commercially and applied in the field for many years. In addition, with the continuous improvement of *Echinococcus* genome data, more high-throughput biomarkers have been identified, and the pathogenesis mechanism is now more comprehensively understood [4]. Recently, it was reported that *Echinococcus* circulating cell-free DNA (cfDNA) in patients’ blood and urine may be used to facilitate accurate differential diagnosis before surgery and could serve as a potential marker for monitoring treatment progress [5]. Yanping Zhao et al. (contribution 4) reported the case of a patient from Sichuan Province, China, who was originally diagnosed as having CE3 by imaging examination, but whose plasma cfDNA sequencing showed 45 reads mapped to the *E. multilocularis* reference genome; thereafter, the case was confirmed as AE via pathological examination after surgery. This report emphasized the application of plasma cfDNA in the preoperative differential diagnosis of CE and AE and its potential to improve the prognosis of patients. Shu-Kun Yang et al. (contribution 5) compared the diagnosis efficiency of native antigen ELISAs with ADAMU-AE/CE (rEm18, rEgB1-based) commercial rapid ICT test kits and imaging techniques in participants exposed to or clinically diagnosed as having CE or AE in northwestern China, and they found that the native antigen ELISAs could not discriminate well between AE and CE. But ADAMU-AE/CE kits showed that they could readily differentiate between cases of CE and AE and showed high specificity, up to 99–100%, indicative of a reliable, accurate, and simple diagnostic tool in clinical settings for the confirmation of suspected CE/AE. On the other hand, native antigen ELISAs detected a significantly higher percentage of active/early stages (types CE1 and CE2) than the ADAMU-CE kit, and are therefore useful in community surveys to understand the exposure level of a population. Elucidating the parasite’s immune evasion mechanisms is necessary in order to explore effective treatment strategies and carry out clinical surveillance. Hui Wang et al. (contribution 6) reported the roles of hepatic dendritic cells in AE patients and mice. They found that greater numbers of dendritic cells (DCs) were present within AE perilesional liver tissues by single-cell RNA sequencing analysis, and a subsequent flow cytometric analysis in the AE mouse model showed that the number of conventional DCs significantly increased after parasite infection. There were different expression levels of molecules CD40, 80, and 86 at early and late stages, indicating a change in the DC phenotype; these findings provide updated information which can be used in the surveillance and control of AE.

In summary, this Special Issue published five research articles and one case report, covering various aspects of CE and AE diagnostics, including PCR, ELISA using recombinant and active antigens, and rapid ICT detection methods. In particular, the cfDNA and LAMP-LFD detection methods demonstrate great potential for more accurate and accessible clinical diagnosis or on-site screening in the future, and thus are worthy of attention. We believe that this Special Issue provides updated information which will enable a more comprehensive understanding of the current advancements in echinococcosis diagnosis, and brings insight which can be used in further research. In the future, it is recommended that a platform for these methods and markers is developed through artificial intelligence modeling and evaluation, so as to render optimal clinical diagnosis, screening, and monitoring strategies for different conditions. From the clinical point of view, sensitive and accurate methods for early diagnosis and postoperative monitoring remain challenging. In addition, to address the need for on-site prevention and control, rapid and simple point-of-care diagnosis methods are yet to be further developed. Compared with that of humans, the detection of animal infections requires further investment, especially with regard to the detection of infection in livestock and wild animals. Traditional autopsy methods are time-consuming and labor-intensive, and the detection results of serum antibodies are not sensitive enough, which means that improving sensitivity should remain on the research agenda. We hope that molecular detection will achieve significant breakthroughs in the future in this regard. Although this Special Issue has not included study reports on environmental DNA (eDNA) testing, from the view of one health control, eDNA is capable of indicating the risk of infection caused by environmental exposure and may provide a new perspective on surveillance tools for disease transmission. Although it is still in its infancy, it is anticipated that eDNA will play a greater role in pathogen monitoring in the near future. Finally, because of the wide epidemic range of *Echinococcus*, multiple species/genotypes, complex life history, and interactions with the hosts, the detection of echinococcosis involves a variety of populations and animal groups. Therefore, efforts should be made to carry out multicenter cooperation and evaluation to simultaneously improve the standardization and reliability of echinococcosis detection.
